# Pr*laeA* Affects the Production of Roquefortine C, Mycophenolic Acid, and Andrastin A in *Penicillium roqueforti*, but It Has Little Impact on Asexual Development

**DOI:** 10.3390/jof9100954

**Published:** 2023-09-22

**Authors:** Yudethzi Marcano, Mariana Montanares, Carlos Gil-Durán, Kathia González, Gloria Levicán, Inmaculada Vaca, Renato Chávez

**Affiliations:** 1 Departamento de Biología, Facultad de Química y Biología, Universidad de Santiago de Chile (USACH), Santiago 9170022, Chile; yudethzi.marcano@usach.cl (Y.M.); cagild@gmail.com (C.G.-D.); kathiavicencio@gmail.com (K.G.); gloria.levican@usach.cl (G.L.); 2 Departamento de Química, Facultad de Ciencias, Universidad de Chile, Santiago 7800003, Chile; mariana.montanares@gmail.com

**Keywords:** asexual development, CRISPR-Cas9, LaeA, *Penicillium roqueforti*, specialized metabolism

## Abstract

The regulation of fungal specialized metabolism is a complex process involving various regulators. Among these regulators, LaeA, a methyltransferase protein originally discovered in *Aspergillus* spp., plays a crucial role. Although the role of LaeA in specialized metabolism has been studied in different fungi, its function in *Penicillium roqueforti* remains unknown. In this study, we employed CRISPR-Cas9 technology to disrupt the *laeA* gene in *P. roqueforti* (Pr*laeA*) aiming to investigate its impact on the production of the specialized metabolites roquefortine C, mycophenolic acid, and andrastin A, as well as on asexual development, because they are processes that occur in the same temporal stages within the physiology of the fungus. Our results demonstrate a substantial reduction in the production of the three metabolites upon disruption of Pr*laeA*, suggesting a positive regulatory role of LaeA in their biosynthesis. These findings were further supported by qRT-PCR analysis, which revealed significant downregulation in the expression of genes associated with the biosynthetic gene clusters (BGCs) responsible for producing roquefortine C, mycophenolic acid, and andrastin A in the ΔPr*laeA* strains compared with the wild-type *P. roqueforti*. Regarding asexual development, the disruption of Pr*laeA* led to a slight decrease in colony growth rate, while conidiation and conidial germination remained unaffected. Taken together, our results suggest that LaeA positively regulates the expression of the analyzed BGCs and the production of their corresponding metabolites in *P. roqueforti*, but it has little impact on asexual development.

## 1. Introduction

The filamentous fungus *Penicillium roqueforti* is an important species within the genus *Penicillium*. This fungus is widely known in the food industry because it is responsible for the ripening of blue-veined cheeses [1,2]. In addition, *P. roqueforti* is an active producer of several specialized metabolites. The study of specialized metabolism in *P. roqueforti* has aroused great interest in recent years, and there are several reviews summarizing our current knowledge on this topic [1,3,4]. 

Roquefortine C, mycophenolic acid, and andrastin A are three of the most known specialized metabolites produced by *P. roqueforti*. Roquefortine C, an alkaloid mycotoxin, is one of the major specialized metabolites produced by *P. roqueforti* [3]. Roquefortine C is considered toxic to humans and animals, and it has been associated with various health risks, including neurotoxicity [1,3]. This mycotoxin is found in cheeses [3], which bears significance in the context of food safety. The biosynthetic gene cluster (BGC) for the biosynthesis of roquefortine C contains four genes, and three of them (namely *rds*, *rpt*, and *rdh*) encode the enzymes required for the biosynthesis of roquefortine C from precursors L-tryptophan, L-histidine, and dimethylallyl diphosphate [5]. Mycophenolic acid is a meroterpenoid molecule widely used (in its salt form) as an immunosuppressant for the prevention of organ transplant rejection [3,6]. It works by inhibiting the enzyme inosine monophosphate dehydrogenase, which is required for the proliferation of T and B lymphocytes, key components of the immune system [3,6]. The BGC for its biosynthesis in *P. roqueforti* contains seven genes [7,8]. Five of these genes (*mpaC*, *mpaDE*, *mpaA*, *mpaH*, and *mpaG*) encode the enzymes that perform the biosynthesis of mycophenolic acid from acetyl-CoA, malonyl CoA, and S-adenosylmethionine [3,9]. Finally, andrastin A is another meroterpenoid produced by *P. roqueforti* [10]. Andrastin A has demonstrated inhibitory effects on farnesyltransferase, an enzyme involved in protein prenylation, and emerges as a promising anticancer compound that has been proposed as a potential natural component of functionalized cheeses [3,11]. The BGC for its biosynthesis in *P. roqueforti* contains 10 genes [12], of which 8 (*adrA*, *adrD*, *adrE*, *adrF*, *adrG*, *adrH*, *adrI*, and *adrK*) encode the enzymes responsible for its production from acetyl-CoA, malonyl CoA, and S-adenosylmethionine [12,13].

The regulation of fungal specialized metabolism is complex and involves multiple regulators [14,15,16,17]. In the case of *P. roqueforti*, the regulators of its specialized metabolism are poorly understood [4]. So far, only three genes encoding global regulators have been analyzed for their effect on specialized metabolism in *P. roqueforti*. They are *pga1*, a gene encoding an α-subunit of a heterotrimeric G protein [18], *sfk1*, a gene encoding a transmembrane protein from the phosphoinositide second messengers pathway [19], and *pcz1*, a protein containing a Zn(II)_2_Cys_6_ domain [20]. At this point, it should be noted that the BGCs responsible for producing roquefortine C, mycophenolic acid, and andrastin A in *P. roqueforti* do not contain genes encoding for cluster-specific transcription factors, which are proteins that specifically regulate the expression of genes within a BGC. Instead, the regulation of these BGCs may be controlled by global regulators [4,14]. 

One of the key global regulators of fungal specialized metabolism is LaeA. It is a nuclear protein with methyltransferase activity, which was originally found in *Aspergillus* spp. [21]. LaeA regulates specialized metabolism in a wide range of fungi from different genera [14,22,23,24]. In *Aspergillus*, LaeA may interact with unknown proteins, or the velvet complex proteins VeA and VelB to regulate the production of specialized metabolites [25]. The exact mechanism of LaeA’s action is still not fully understood, but it is thought that this protein controls specialized metabolism by modifying the chromatin structure in genomic regions containing BGCs. This modification is believed to affect the expression of genes involved in fungal specialized metabolism [26,27,28,29]. In line with this role, several genes directly regulated by LaeA or the LaeA/VeA complex have been recently identified, including genes associated with specialized metabolism [25].

In addition to its role in regulating specialized metabolism, LaeA has been shown to play a role in asexual fungal development, particularly in conidiation, growth, and conidial germination [30,31,32,33,34]. Indeed, in *A. nidulans*, LaeA has been found to directly target genes that encode important regulators of asexual development, such as *flbA*, *flbC*, and *trxA* [25].

The role of LaeA has been analyzed in several fungi from the genus *Penicillium*. In *P. chrysogenum* (currently reclassified as *P. rubens* [35]), LaeA positively regulates conidiation, penicillin-gene expression, and penicillin production [30,36,37]. In *P. expansum*, LaeA appears to positively regulate patulin gene expression, patulin synthesis, and conidiation [38,39]. In *P. oxalicum*, the deletion of *laeA* impaired conidiation and downregulated the expression of several BGCs [40], while in *P. digitatum*, the deletion of this gene resulted in a marked decrease in conidiation, a slight inhibition of apical growth, and a decreased expression of three BGCs including the one involved in tryptoquialanine biosynthesis [41]. In *P. citrinum*, Baba et al. [42] showed that the disruption of *laeA* resulted in faster hyphal growth and reduced production of aerial mycelium compared with the wild-type strain. Furthermore, they found that *laeA* positively controls the biosynthesis of mevastatin ML-236B in this fungus [42]. On the other hand, in *P. dipodomyis*, the overexpression of *laeA* induced the production of several sorbicillinoids and changed spore morphology [43], whereas in *P. brocae*, its overexpression led to the production of four specialized metabolites: spinulosin, pyranonigrin F, fumigatin chlorohydrin, and iso-fumigatin chlorohydrin [44]. Therefore, although the specific effects of LaeA may differ between species, the studies mentioned suggest that LaeA plays a crucial role in regulating specialized metabolism and asexual development in *Penicillium* fungi. 

Considering the scarce knowledge about the regulation of the biosynthesis of specialized metabolites in *P. roqueforti* [4] and especially the lack of studies about the role of LaeA in this fungus, in this work we decide to disrupt *laeA* in *P. roqueforti* and evaluate the effect of the gene disruption on the production of the specialized metabolites roquefortine C, mycophenolic acid, and andrastin A, as well as on asexual development. 

## 2. Materials and Methods

### 2.1. Fungal Strain and Culture Media

*Penicillium roqueforti* strain CECT 2905 was used for all the experiments in this study. The fungus was routinely maintained on potato dextrose agar (PDA) and incubated at 28 °C. YES agar [12] was used for specialized metabolite production and RNA extractions. To characterize the asexual development phenotype, CYA, Czapek, CM [45], and Power [46] media were additionally employed. The culture conditions for these purposes are detailed below.

### 2.2. Search for the PrlaeA Gene in P. roqueforti CECT 2905 and Sequence Analyses

The *laeA* gene from the closely related species *P. rubens* (GenBank accession EU685842; [30]) was used to search for the *laeA* gene in the genome of *P. roqueforti* CECT 2905 [47] by BLASTN and BLASTX. The gene found and its deduced protein were named Pr*laeA* and PrLaeA, respectively (see Section 3). PrLaeA was submitted to sequence analyses. Analysis of conserved domains was conducted using CDD at NCBI [48], while multiple sequence alignments were performed using Clustal Omega [49] at the EMBL-EBI web service (https://www.ebi.ac.uk/Tools/msa/clustalo/, accessed on 16 May 2023). 

### 2.3. Design of a Cassette for the Disruption of PrlaeA by CRISPR-Cas9 Technology

The Pr*laeA* gene in *P. roqueforti* CECT 2905 was disrupted by CRISPR-Cas9 technology following the protocol of Seekles et al. [50], with minor modifications. A target sequence was selected using the CHOPCHOP program (https://chopchop.cbu.uib.no/, accessed on 29 August 2021; [51]), which contains the *P. roqueforti* genome in its database. CHOPCHOP was used with default parameters. The protospacer was chosen based on its location (near the 5´end of the gene), efficiency, and the absence of off-target sites. The specificity of the target sequence predicted by CHOPCHOP (5′-CTCCAGATACTATTCACACT-3′) was verified by aligning it against the entire genome of *P. roqueforti* CECT 2905 using BLASTN.

After obtaining the target sequence, a cassette was designed for its expression. The cassette was exactly as described by Seekles et al. [50]. Briefly, the cassette contained the promoter and gene sequences of proline-tRNA (tRNA^Pro1^) from *A. niger*, a hammerhead ribozyme sequence, the target sequence, a suitable crRNA, and the terminator of tRNA^Pro1^. The cassette also included *Pac*I restriction sites at both ends for cloning. The cassette was 377 bp in size and was synthesized by Integrated DNA Technologies (IDT, Coralville, IA, USA).

### 2.4. Construction of Plasmid pCPrlaeA, Transformation of P. roqueforti and Selection of Transformants

The cassette was digested with *Pac*I and cloned into plasmid pFC333 [52] previously digested with *Pac*I. Plasmid pFC333 contains the gene encoding for the Cas9 enzyme under the control of the *tef1*-promoter from *A. nidulans*, the AMA region for autonomous replication of the plasmid, and the phleomycin-resistant marker for transformants selection [52]. After cloning, the resulting plasmid was named pCPrlaeA. Next, pCPrlaeA was used for the transformation of *P. roqueforti*, following the protocol described by Chávez et al. [53]). Homokaryotic strains were obtained by seeding serial dilutions of transformant conidia onto a Czapek medium supplemented with phleomycin. To induce the loss of phleomycin resistance, transformants were transferred between four and eight times to non-selective growth conditions (PDA without phleomycin). Several transformants lost phleomycin resistance, as confirmed by the absence of growth of transformants on selective media (see Results).

Finally, the disruption of Pr*laeA* in transformants was confirmed by sequencing. For this purpose, DNA was extracted from transformants using the method described by Gil-Durán et al. [45] and used as a template for amplification of the target region of Pr*laeA* using primers Conf-LaeA-CRISPR-FW and Conf-LaeA-CRISPR-RV (Table 1). The amplified regions were cloned into pGEM-T Easy and sequenced by Macrogen Inc. (Seoul, Republic of Korea).

### 2.5. Extraction of Specialized Metabolites and HPLC Analyses

To produce specialized metabolites, *P. roqueforti* strains were cultured on YES agar at 28 °C for 15 days. The extraction and HPLC analyses of roquefortine C, mycophenolic acid, and andrastin A were performed following the protocols described by Torrent et al. [19], Del-Cid et al. [7], and Rojas-Aedo et al. [12], respectively. The production of these specialized metabolites was normalized by the dry weight of fungal mycelia, as described by García-Rico et al. [18].

### 2.6. Measurement of Gene Expression by qRT-PCR 

To measure gene expression, RNA was extracted from *P. roqueforti* strains grown on YES agar for 15 days at 28 °C as described previously [45]. The extracted RNA was used for qRT-PCR analyses of key genes from the BGCs responsible for producing roquefortine C, mycophenolic acid, and andrastin A in *P. roqueforti*. The qRT-PCR protocols for analyzing these genes have been previously described [7,12,20]. Primers used for these purposes are detailed in Table 1. Gene expression levels were normalized to the β-tubulin gene and analyzed using the 2^−ΔΔCt^ method [54]. 

### 2.7. Measurement of Colony Growth Rate, Conidial Production and Conidial Germination 

The characterization of the asexual development phenotype was conducted following the methodology described by Gil-Durán et al. [45], with slight modifications. For determining the colony growth rate, solid agar plates containing different media were inoculated in the center with 0.2 μL of a conidial suspension (1 × 10^6^ conidia) and incubated at 28 °C. The colony diameter was measured daily, and the colony growth rate was calculated using linear regression analysis of the colony diameter over time. 

For assessing conidial production, a conidial suspension of 5 × 10^5^ conidia/mL (100 μL) was inoculated onto Petri dishes containing a Power medium, which is optimized for sporulation [46]. The dishes were then incubated at 28 °C for 1, 3, 5, or 7 days. Conidia produced were collected by adding NT solution (0.9% NaCl, 0.05% Triton) and scraping the surface of the plate with an inverted Pasteur pipette. This process was repeated once. The obtained conidia were quantified using a Neubauer chamber, and the values were expressed as conidia/mm^2^ of surface area.

To measure conidial germination, flasks containing CM medium with a conidial concentration of 2 × 10^5^ conidia/mL were incubated at 28 °C. At regular intervals, 10 μL samples were taken, observed under a microscope, and the number of germinated and non-germinated conidia was counted in 10 randomly selected fields. Conidia were considered germinated when the length of their germ tubes equaled or exceeded the diameter of the conidia. The data were plotted as the percentage of germination versus time. 

## 3. Results

### 3.1. Analysis of the PrlaeA Gene and Its Deduced Protein from P. roqueforti

**Figure 1:**
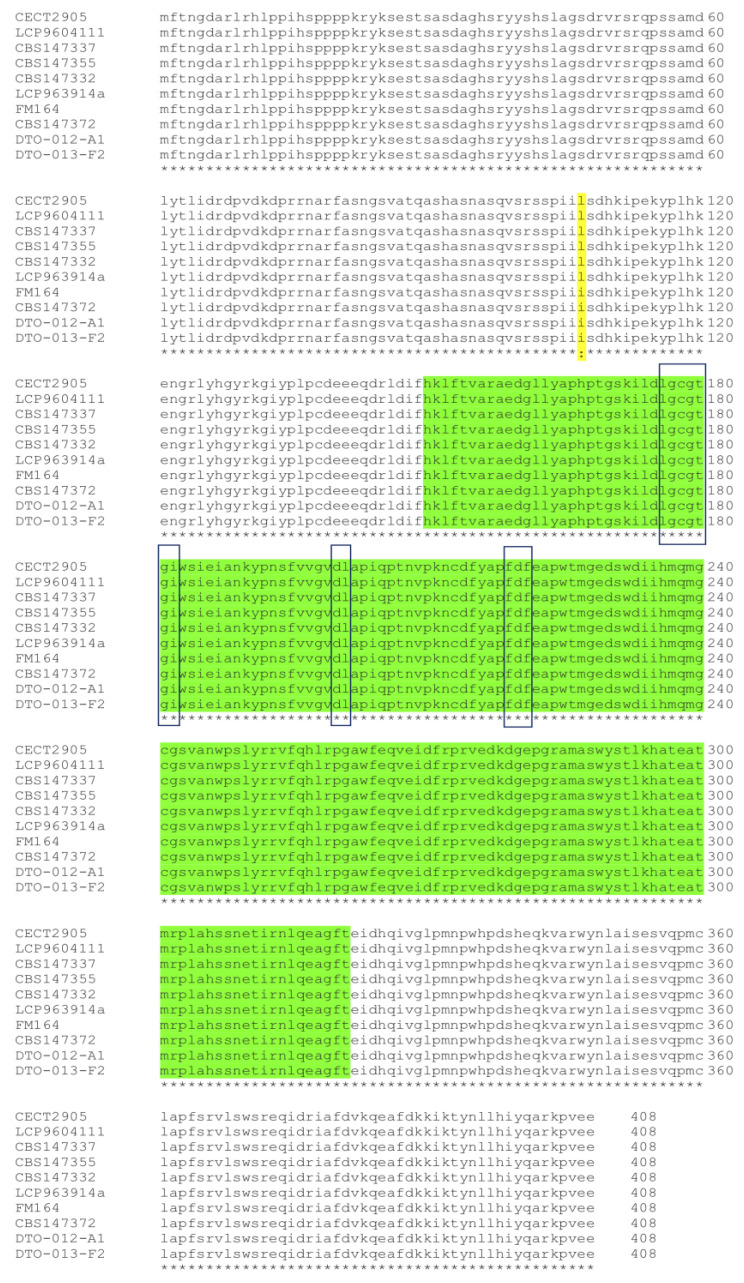
Multiple alignments of LaeA proteins from various strains of *P. roqueforti*. Each strain code is labeled on the left of its corresponding sequence. The aligned sequences exhibit overall identity with a single conservative change (I or L) at position 107 (highlighted in yellow). The methyltransferase domain (residues 150 to 321) is emphasized in green. The three conserved SAM binding sites, located at positions 176–182, 200–201, and 219–221, are boxed. Asterisks (*) denote positions with a single, fully conserved residue, while semi-colons (:) indicate positions with highly conserved residues.

To identify the Pr*laeA* gene in *P. roqueforti* CECT 2905, the *laeA* gene sequence from *P. rubens* [30] was utilized to perform BLASTN and BLASTX scans of the *P. roqueforti* CECT 2905 genome [47]. The search led to the identification of the Pr*laeA* gene, which is located on contig MSQC01000647.1 and spans nucleotides 44,522 to 45,800. The gene is 1279 bp long and contains one intron. The gene encodes a protein (PrLaeA) of 408- amino acids that is highly conserved (99–100% identity) in different *P. roqueforti* strains (Figure 1). The analysis of conserved domains of PrLaeA revealed the presence of a methyltransferase domain containing conserved S-adenosylmethionine (SAM) binding sites (Figure 1), which are characteristic of LaeA proteins found in filamentous fungi [55]. 

### 3.2. Disruption of PrlaeA Gene in P. roqueforti CECT 2905 by CRISPR-Cas9

**Figure 2:**
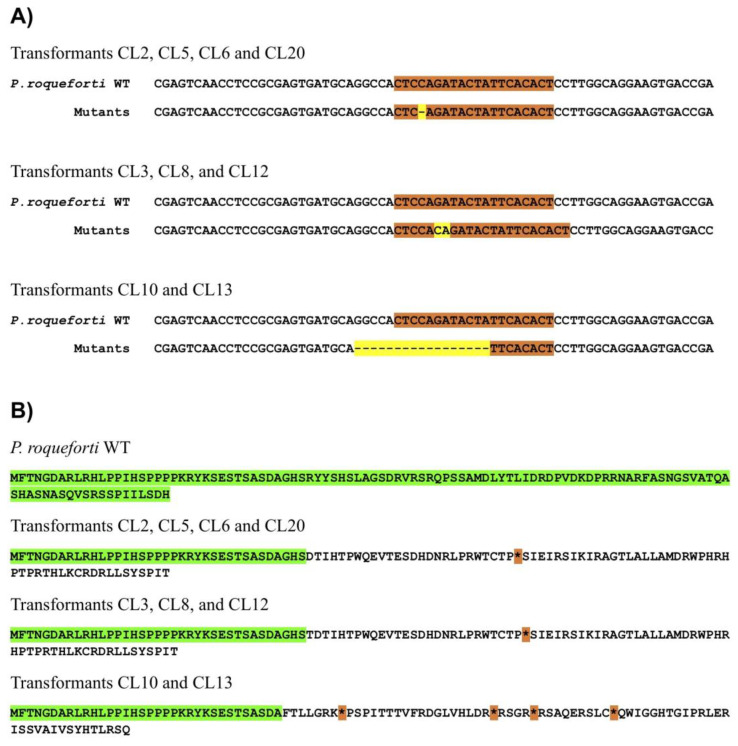
(**A**) Comparison of the nucleotide sequences of the target region of Pr*laeA* in *P. roqueforti* wild-type (labeled as *P. roqueforti* WT) and disrupted transformants. The target regions are marked in orange, while the CRISPR-Cas9-induced insertions or deletions are highlighted in yellow in the transformants. (**B**) Analysis of the amino end of the PrLaeA protein from *P. roqueforti* wild-type and transformants. In transformants, the initial segment of PrLaeA (35–37 amino acids) remains unchanged (highlighted in green), but subsequent regions exhibit frameshifts and premature stop codons (asterisks highlighted in orange).

To disrupt the Pr*laeA* gene in *P. roqueforti* CECT 2905, plasmid pCPrlaeA was introduced into the fungus, and 27 phleomycin-resistant transformants were obtained. These transformants were then cultured several times in phleomycin-free PDA media, resulting in 14 transformants losing phleomycin resistance. Next, the target regions of Pr*laeA* in these 14 transformants were sequenced, revealing that 9 transformants had deletions or insertions of bases in the target region, leading to a loss of protein frameshift and a production of premature stop codons, effectively disrupting the Pr*laeA* gene (Figure 2). Specifically, one-base deletions were observed in four transformants (CL2, CL5, CL6, and CL20), two-base insertions were found in three transformants (CL3, CL8, and CL12), and a large deletion of seventeen bases was detected in two transformants (CL10 and CL13) (Figure 2). According to these results, we selected one transformant from each genotype (CL6, CL8, and CL13) randomly for further experiments.

### 3.3. The Disruption of PrlaeA Reduces the Production of Roquefortine C, Mycophenolic Acid, and Andrastin A in P. roqueforti

**Figure 3:**
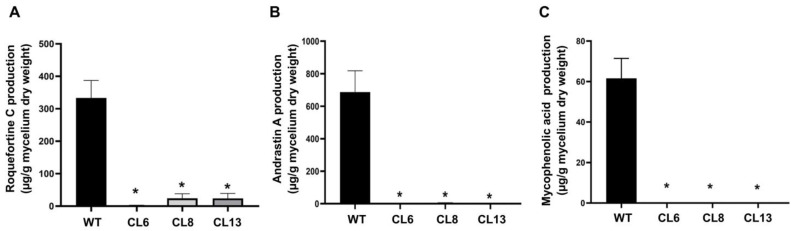
Production of roquefortine C (**A**) andrastin A (**B**) and mycophenolic acid (**C**) by Pr*laeA*-disrupted transformants CL6, CL8, and CL13 compared with control *P. roqueforti* wild-type strain (WT). The data represent the average values from three replicas of three independent experiments. Error bars indicate the standard deviation of the mean value. Statistically significant differences between the transformants and the wild-type strain are denoted by asterisks (*p* < 0.05, determined using Student’s *t*-test).

To investigate the impact of Pr*laeA* disruption on specialized metabolism in *P. roqueforti*, we quantified the production of three of the main specialized metabolites produced by this fungus, namely roquefortine C, mycophenolic acid, and andrastin A. As shown in Figure 3, the production of these metabolites was significantly reduced in all tested transformants compared with the wild-type strain. Specifically, while the wild-type strain produced approximately 333 μg/g of roquefortine C, the transformants produced only between 2.5 and 23.6 μg/g, indicating a reduction of between 93 and 99% in the production of this compound, depending on the transformant analyzed. In the case of andrastin A, the wild-type strain produced 686 μg/g of the compound, whereas the production of the transformants ranged from 1.5 to 2.3 μg/g, representing a reduction of 99.7 to 99.8% in the production of andrastin A. Finally, the production of mycophenolic acid was undetectable in the transformants under the analyzed conditions, while *P. roqueforti* CECT 2905 produced 61 μg/g of the compound. 

### 3.4. The Lower Production of Specialized Metabolites in Disrupted Strains of P. roqueforti Correlates with the Downregulation in the Expression of Key Genes Involved in Their Biosynthesis

To examine the role of Pr*laeA* in the transcription of genes involved in the biosynthesis of roquefortine C, andrastin A, and mycophenolic acid in *P. roqueforti*, we conducted qRT-PCR experiments. For this purpose, we focused on three key genes from each BGC encoding biosynthetic enzymes. For roquefortine C biosynthesis, we quantified the expression of *rds*, which encodes a non-ribosomal peptide synthase and serves as the initial enzyme in roquefortine C biosynthesis [5]. Additionally, we assessed *rdh*, responsible for encoding a dehydrogenase that acts on the diketopiperazine or the intermediate roquefortine D, and *rpt*, which encodes an enzyme in charge of the prenylation of roquefortine D or the reduced diketopiperazine [5]. In the case of andrastin A biosynthesis, we analyzed *adrD*, responsible for the first reaction in andrastin A biosynthesis and encoding a polyketide synthase [12]. We also examined *adrH*, which encodes an FAD-dependent monooxygenase responsible for the formation of epoxyfarnesyl-DMOA methyl ester from the precursor farnesyl-DMOA methyl ester, and *adrI*, which encodes a terpene cyclase catalyzing the cyclization of an epoxyfarnesylated precursor to yield andrastin D, the first andrastin in the pathway [12,13]. Finally, for mycophenolic acid biosynthesis, we measured the expression of *mpaC*, responsible for the first reaction in mycophenolic acid biosynthesis and encoding a polyketide synthase [7]. We also assessed *mpaH*, which encodes an enzyme mediating the oxidative cleavage of a farnesylated precursor of the intermediate demethylmycophenolic acid [7,9], and *mpaG*, encoding an O-methyltransferase catalyzing the methylation of demethylmycophenolic acid to form mycophenolic acid [4,7]. 

**Figure 4:**
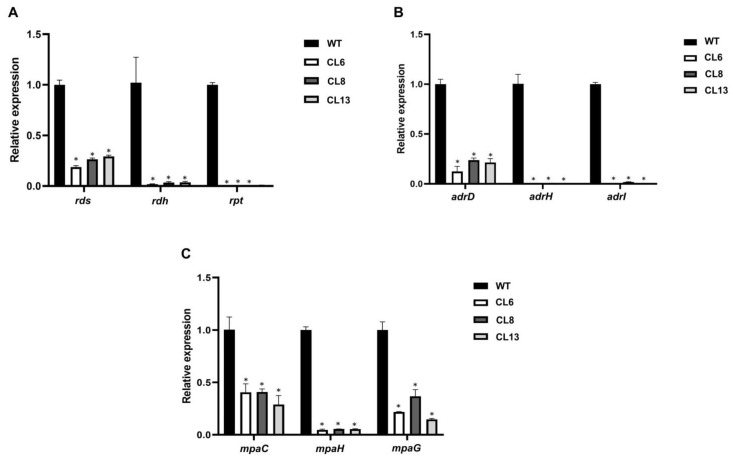
qRT-PCR analyses of the expression of genes related to roquefortine C (**A**) andrastin A (**B**) and mycophenolic acid (**C**) biosynthesis in Pr*laeA*-disrupted transformants (CL6, CL8, and CL13) compared with the control *P. roqueforti* wild-type strain (WT). Three genes of each BGC were analyzed, namely *rds*, *rdh*, and *rpt* from the roquefortine C BGC, *adrD*, *adrH*, and *adrI* from the andrastin A BGC, and *mpaC*, *mpaH*, and *mpaG* from the mycophenolic acid BGC. The data represent the average of three replicates from three independent experiments. Error bars represent the standard deviation of the mean. The symbol * denotes statistically significant differences between transformants and the wild-type strain (*p* < 0.05, Student’s *t*-test).

Figure 4 demonstrates that the disruption of Pr*laeA* led to reduced expression levels of all biosynthetic genes analyzed in comparison to the wild-type strain of *P. roqueforti*. For roquefortine C biosynthesis, and depending on the specific transformant under analysis, the expression levels of the biosynthetic genes *rds*, *rdh*, and *rpt* ranged from 19% to 29%, 1.8% to 3.3%, and 0.4% to 0.9%, respectively, when compared with the levels observed in the wild-type fungus (Figure 4A). In the case of andrastin A biosynthesis, the expression levels of *adrD*, *adrH*, and *adrI* ranged from 12% to 24%, 0.1% to 0.7%, and 0.3% to 1.9%, respectively, compared with the levels observed in the wild-type strain (Figure 4B). Finally, the expression levels of the biosynthetic genes *mpaC*, *mpaH*, and *mpaG* for mycophenolic acid ranged from 29% to 41%, 4.6% to 5.5%, and 15% to 22% respectively, in comparison to the levels observed in the wild-type fungus (Figure 4C).

### 3.5. The Inactivation of PrlaeA Has Little Impact on Asexual Development in P. roqueforti

**Figure 5:**
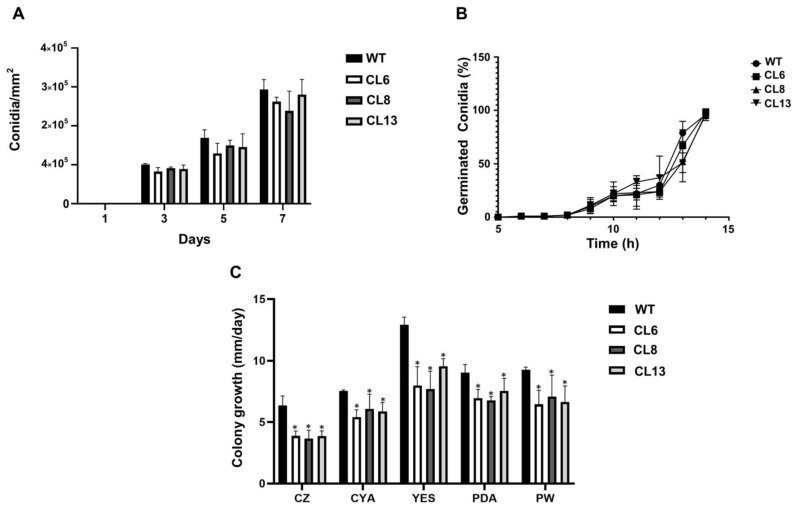
Characterization of the asexual development phenotype in Pr*laeA*-disrupted transformants CL6, CL8, and CL13 compared with the control *P. roqueforti* wild-type strain (WT). (**A**) Conidial production in Petri dishes containing a Power medium, expressed as conidia/mm^2^ of the surface. Error bars represent the standard deviation of three replicas from three independent experiments. No statistically significant differences in conidial production were observed among all strains. (**B**) Analysis of germination kinetics of *P. roqueforti* strains in a CM liquid medium, expressed as the percentage of germinated conidia over hours of incubation. Error bars represent the standard deviations of three replicates in three independent experiments. No differences were observed among the strains. (**C**) Colony growth rates (mm/day) of *P. roqueforti* strains on Czapek (CZ), CYA, YES, PDA, and Power (PW) media. Error bars represent the standard deviation of three replicas from three different experiments. The symbol * indicates statistically significant differences compared with the WT strain (*p* < 0.05, Student’s *t*-test).

In addition to its role in specialized metabolism, LaeA has also been implicated in asexual development in several fungi [25,30,31,32,33,34]. Therefore, it was of interest to investigate the role of Pr*laeA* in the asexual development of *P. roqueforti*. As shown in Figure 5, the inactivation of Pr*laeA* did not have any significant effect on either conidiation or conidial germination. For both parameters, the transformants have similar behaviors in comparison with the wild-type strain. However, we observed a slightly slower apical growth in the transformants compared with the wild-type strain in several media tested. Depending on the specific media used, transformants displayed a growth rate ranging between 57.6% and 83.4% of the wild-type fungus. Therefore, we conclude that the inactivation of Pr*laeA* has little effect on asexual development in *P. roqueforti*.

## 4. Discussion

Compared with other fungi, our current understanding of the regulation of specialized metabolism in *P. roqueforti* is limited [4]. The present work makes a significant contribution to this topic by examining the impact of the regulator LaeA on the production of important specialized metabolites of this fungus.

Until recently, genetic studies of *P. roqueforti* were hampered by the lack of efficient tools for gene disruption [4]. As a result, most studies investigating the genetics of specialized metabolism in this fungus have employed alternative methods, mainly RNAi-mediated gene silencing technology [5,7,8,12,19,20,30]. Recently, Seekles et al. [50] developed the first CRISPR-Cas9 system dedicated to *P. roqueforti*, opening new opportunities for studying the genetics of this fungus. This system has previously been successfully used for gene disruption [50,56] and here, we have utilized the same approach to examine the role of the important regulator LaeA.

Our results indicate that PrLaeA plays a crucial role in regulating the specialized metabolism of *P. roqueforti*, which in general terms, is consistent with findings in other fungi [14,21,22,23,24]. However, we also observed important differences in our results compared with prior research, which are discussed below.

Our data indicate that Pr*laeA* is a positive regulator of roquefortine C production. Specifically, our findings demonstrate that the disruption of Pr*laeA* significantly decreases the production of this compound in *P. roqueforti*, which is accompanied by a reduction in the expression of the three genes responsible for roquefortine C biosynthesis. Our results are partially consistent with the findings of Kumar et al. [38] who investigated the role of *laeA* in *P. expansum*. They evaluated the role of *laeA* in this fungus by assessing the expression of 54 specialized metabolism genes in wild-type and Δ*laeA* strains under various conditions. The authors identified several genes positively regulated by *laeA* in *P. expansum*, including those belonging to the roquefortine C BGC [38]. Unfortunately, they did not measure the levels of roquefortine C in culture to establish a correlation between the gene expression and the compound production.

In contrast, our results differ from those reported for *P. oxalicum* and *P. chrysogenum* regarding the regulation of roquefortine C biosynthesis by *laeA*. In *P. oxalicum*, Zhang et al. [40] studied the expression of 28 BGCs (including the roquefortine C BGC) in a Δ*laeA* background. Although they identified several BGCs affected by the deletion of *laeA*, the BGC responsible for roquefortine C biosynthesis was not influenced. In the case of *P. chrysogenum*, *laeA* has a clear effect on the production of penicillin, but it does not regulate the biosynthesis of roquefortine C [30]. Specifically, Kosalková et al. [30] observed that the levels of roquefortine C were comparable between a *laeA* knockdown mutant and the wild-type strain of *P. chrysogenum*. This correlated with the absence of differences in the expression of *rds*, the first gene involved in roquefortine C biosynthesis, between the two *P. chrysogenum* strains [30]. Collectively, our findings, along with those of previous studies, suggest that the response of the roquefortine C BGC to *laeA* in the genus *Penicillium* is dependent on the species. Thus, in species such as *P. roqueforti* and *P. expansum*, LaeA exerts a positive effect on the roquefortine C BGC, whereas in other species such as *P. oxalicum* and *P. chrysogenum*, the roquefortine C BGC appears to be insensitive to LaeA. It has been proposed that differences in the regulation of the roquefortine C BGC may be attributed to factors such as the chromosomal location of the roquefortine BGC in each genome, or the chromatin structure of the promoters of genes involved in its biosynthesis [23,30]. Further work is required to identify the underlying causes of these species-specific differences in the biosynthesis of roquefortine C in response to LaeA.

In *P. roqueforti*, we found that disrupting Pr*laeA* significantly reduced andrastin A production and downregulated the expression of key genes from the andrastin A BGC, suggesting a positive role for Pr*laeA* in andrastin A biosynthesis. These findings contrast with those observed in *P. expansum*. As previously mentioned, Kumar et al. [38] evaluated the expression of various specialized metabolism genes in this fungus, including those related to the andrastin A biosynthesis, in a Δ*laeA* background. Under the conditions tested by these authors in *P. expansum*, the andrastin A BGC was not regulated by LaeA. Similar to roquefortine C, these findings, combined with our results in *P. roqueforti*, suggest that the influence of LaeA on andrastin A BGC might vary depending on the species. Nevertheless, to establish a conclusive correlation between LaeA and andrastin A production it is necessary to investigate the role of *laeA* in other fungi that produce this compound.

Mycophenolic acid is synthesized by several fungi, particularly in the genus *Penicillium* [57]. Recent studies have shed light on the molecular mechanisms involved in the biosynthesis of mycophenolic acid in *P. brevicompactum* and *P. roqueforti* [7,8,58]. Regarding the regulation of mycophenolic acid production, several studies have indirectly addressed this topic by exploring the influence of different culture conditions on the production of this compound by fungi [59,60,61,62,63]. Furthermore, various studies have addressed the development of over-producing fungal strains through mutagenesis methods [59,61,63,64,65]. At the molecular level, the effect of two regulators (*sfk1* and *pcz1*) on the production of mycophenolic acid in *P. roqueforti* has been studied [19,20]. However, to the best of our knowledge, the effect of *laeA* on mycophenolic acid production has not been investigated in any fungus before the current study. Our findings indicate that the inactivation of Pr*laeA* in *P. roqueforti* leads to the downregulation of key genes involved in mycophenolic acid biosynthesis, and significantly reduces the production of this compound, suggesting a positive regulatory role of PrLaeA in mycophenolic acid production. Future investigations will be necessary to determine whether this regulatory effect of *laeA* is conserved in other important mycophenolic acid producers, such as *P. brevicompactum*, *P. stoloniferum*, and *P. echinulatum*.

Our findings regarding the role of Pr*laeA* in regulating the biosynthesis of roquefortine C, andrastin A, and mycophenolic acid production in *P. roqueforti* represent a significant advancement and set the stage for further investigation into the regulatory mechanisms governing both primary and specialized metabolism in this fungus. We propose that these future investigations should incorporate techniques such as metabolomic analysis using LC-HRMS, RNA-seq to identify genes differentially expressed by Pr*laeA*, proteomic analysis for the identification and quantification of proteins regulated by Pr*laeA*, and chromatin immunoprecipitation experiments for identifying genomic regions directly bound by this regulator. The integration of these methodologies will provide deeper insights into the role of Pr*laeA* in the primary and specialized metabolism of *P. roqueforti*, significantly advancing our understanding of the role of this regulator.

As previously mentioned, LaeA has been implicated in various aspects of asexual development in different fungi. In the case of Pr*laeA*, we found that its disruption in *P. roqueforti* had no impact on asexual development, except for a slight decrease in apical growth. The role of LaeA in asexual development can vary among different fungal species. Regarding apical growth, our findings are consistent with previous studies in *P. digitatum* and *P. expansum* strain Is-Pe-21, where disruption of *laeA* resulted in decreased apical growth [38,41]. However, our findings differ from those in *P. citrinum*, where the deletion of *laeA* was found to enhance hyphal growth [42]. Regarding conidial germination, the effects of *laeA* deletion can be positive, negative, or null, depending on the fungal species analyzed [32,34,41]. In our case, we did not observe a significant difference in conidial germination rate after disrupting Pr*laeA* in *P. roqueforti*, in accordance with previous findings in *P. digitatum* [41]. Finally, in terms of conidiation, the role of LaeA is highly variable among different fungi [31,32,34,66]. In *Penicillium* species, LaeA has been demonstrated to positively regulate conidiation in *P. chrysogenum*, *P. expansum*, *P. oxalicum*, and *P. digitatum* [37,38,40,41]. However, in contrast to these results, our study revealed that the disruption of Pr*laeA* had no significant effect on conidiation in *P. roqueforti*. Thus, our findings differ from those observed in other *Penicillium* species and are more in line with what was observed in *A. flavus* [66]. The varying phenotypes of *laeA* mutants in different fungal species underscore the idea that LaeA plays diverse roles in asexual development across fungi. Recent studies conducted in *Aspergillus* support this notion, demonstrating that LaeA interacts with several genes responsible for encoding proteins involved in asexual development. For instance, a study on *A. nidulans* identified 1834 potential direct target genes of LaeA, including key developmental regulators like *flbB* and *flbC*, known activators of asexual spore formation, and *stuA*, a spatial modifier of conidiophore morphogenesis [25]. Another investigation, focusing on *A. fumigatus*, revealed that LaeA epigenetically regulates the expression of *brlA*, a gene encoding a transcription factor pivotal in regulating conidiophore development [29]. Despite these advances, the intricate network of interactions between LaeA and asexual development regulators remains incompletely understood. Therefore, further investigations are necessary to unravel the mechanisms of the interplay between LaeA and regulators of asexual development across diverse fungal species. 

## Figures and Tables

**Table 1:** Primers used in this work.

Name of the Primer	Sequence (5′---3′)	Used for:	Reference
Conf-LaeA-CRISPR-FW Conf-LaeA-CRISPR-RV	ATGTTTACGAACGGGGAT AAAGCGAGCGTTCCTGC	Amplification of target sequence of Pr*laeA* gene	This work
RoqA-qpcr-Fw RoqA-qpcr-Rv	ATCTGTGGCACGATTCATCA CTCGACCCTGACCATTGTTT	*rds* gene expression analysis by qRT-PCR	[20]
RoqR-qpcr-Fw RoqR-qpcr-Rv	TATGCCTTCAAGGGTGGTCT TTGAAGTTAGCCCAGCGAGT	*rdh* gene expression analysis by qRT-PCR	[20]
RoqD-qpcr-Fw RoqD-qpcr-Rv	AAAGGTTGAGGAGCACTGGA AACTCCACCCACAACTCTCG	*rpt* gene expression analysis by qRT-PCR	[20]
adrD-qPCR-fw adrD-qPCR-rv	GGCTCGGACGACTATACTGA AGTACAGAACGCCTGGAGTG	*adrD* gene expression analysis by qRT-PCR	[12]
adrH-qPCR-fw adrH-qPCR-rv	GACACCCAATATCGGACAAG AAGGCATCTGCGTGAACTAC	*adrH* gene expression analysis by qRT-PCR	[12]
adrI-qPCR-fw adrI-qPCR-rv	ACGTCGCGAAAAGACAAGAT TCGCGGTTGGGTAGATAAAG	*adrI* gene expression analysis by qRT-PCR	[12]
mpaC-qPCR-FW mpaC-qPCR-RV	CAGGGGTTCTGTGTGGGTAT AATACAGACAGCGAGCCGTA	*mpaC* gene expression analysis by qRT-PCR	[7]
mpaG-qPCR-FW mpaG-qPCR-RV	CGGGTAAGGGGATAGATTGT TCACATTCATAGCCACGAGA	*mpaG* gene expression analysis by qRT-PCR	[7]
mpaH-qPCR-FW mpaH-qPCR-RV	CCGCTGATACTACTGCCACT GCATTGAAGTTCTGCCGTAT	*mpaH* gene expression analysis by qRT-PCR	[7]
qRT-btub-fw qRT-btub-rv	TCCAAGGTTTCCAGATCACC GAACTCCTCACGGATCTTGG	β-tubulin gene expression analysis by qRT-PCR	[7]

## Data Availability

Not applicable.
